# Circulating calprotectin as a supporting inflammatory marker in discriminating SARS-CoV-2 infection: an observational study

**DOI:** 10.1007/s00011-021-01465-y

**Published:** 2021-05-06

**Authors:** Fabio Cherubini, Antonio Cristiano, Alessandra Valentini, Sergio Bernardini, Marzia Nuccetelli

**Affiliations:** 1 Department of Experimental Medicine, University of Tor Vergata, Via Montpellier, 1, 00133 Rome, Italy; 2 Department of Biomedicine and Prevention, Tor Vergata University, Rome, Italy; 3 Tor Vergata University Hospital, Rome, Italy; 4 IFCC Emerging Technologies Division, Milan, Italy

**Keywords:** Circulating calprotectin, SARS-CoV-2, COVID-19, Inflammatory marker, Laboratory medicine

## Abstract

**Objective and design:**

Fecal calprotectin (CLP) is widely known for its detection in stools of patients with inflammatory bowel diseases (IBDs), to investigate the intestinal inflammatory status. Current research is promoting the circulating protein role as a systemic inflammatory marker. However, most studies report serum calprotectin analysis although plasma assay prevents its massive release by granulocytes. In this perspective, the ongoing SARS-CoV-2 pandemic deserves deployment of convenient and easy-to-dose markers that could reliably address the state of infection.

**Methods:**

We analyzed serum circulating calprotectin (cCLP) levels in hospitalized COVID-19 patients and plasma cCLP levels from patients with suspected SARS-CoV-2 infection, then assessed negative or positive on molecular tests.

**Results:**

Our results confirm a significant circulating calprotectin increase in infected subjects respect to controls, in serum and plasma. Moreover, plasma calprotectin has higher levels in suspected patients with positive SARS-CoV-2-RT-PCR, compared to suspected patients with negative SARS-CoV-2-RT-PCR. Furthermore, ROC curves results showed the circulating plasma calprotectin discriminatory ability to differentiate infected SARS-CoV-2 patients at a cutoff value greater than 131.3 ng/ml.

**Conclusions:**

Our data propose circulating calprotectin as a new, quantitative and predictive marker, which in addition to being an interesting generic inflammatory marker may provide important indications in SARS-CoV-2 infection.

## Introduction

Calprotectin (CLP) is a calcium and zinc-finger heterodimer (36.5 kDa) formed by proteins S1008 and S1009. It belongs to S100 calcium-binding protein family comprising small acidic proteins with high solubility in 100% ammonium sulfate solution; thus the name S100 [1]. CLP has different synonyms. It was first called leukocyte protein L1 [2]; current names are myeloid-related-proteins-8 and  – 9 (MRP8, MRP9) owed to abundance in neutrophils, monocytes and early-differentiated macrophages [3], where it represents 5% of total proteins and 60% of cytosolic proteins, respectively, and calgranulin (A and B) linked to distinctive calcium-binding properties in granulocytes [4].

CLP is a potent antimicrobial protein: an initial pro-inflammatory stimulus, triggers its production via Toll-Like Receptor 4 (TLR4) expressed on granulocytes [5]. CLP release prevents bacterial growth by impairment of zinc-dependent enzymes upon zinc (Zn) sequestration. Indeed, mounting evidence proves CLP effect against several bacterial species such as *Escherichia coli* and *Staphylococcus aureus* among others, along with fungistatic function [6, 7].

CLP involvement in inflammatory process is broadly known: first granulocytes increase CLP expression and secretion upon inflammatory stimuli, then CLP acts as a cytokine-like protein by binding surface receptors and triggering pathways engaged in mounting immune response. Conversely, CLP exerts an immune-regulatory role by chelation of Zn divalent cations, thus resulting in zinc-dependent metallo-proteinases inhibition, responsible for activation of pro-inflammatory cytokines such as tumor necrosis factor (TNF) [8]. Furthermore, CLP drives several cellular processes via calcium homeostasis regulation, such as rearrangement of cytoskeletal components, cell cycle progression, proliferation, migration and survival. Collective data agree with the idea of CLP as a smart molecule in which its function outcome depends on concentration and intra- or extracellular localization [9].

CLP is found in various body fluids such as serum and synovial liquid in proportion to severity of any existing inflammation. Indeed, serum levels are usually reported below 1 μg/ml in healthy subjects, but during inflammation they may increase by 100 times [10]. Besides, healthy subjects’ concentration in feces is about six times that of normal plasma [11]. Therefore, CLP promptly became a reliable biomarker in inflammatory diseases including inflammatory bowel diseases (IBDs), rheumatoid arthritis, cystic fibrosis, chronic bronchitis, many types of cancers and neurodegenerative disorders, such as Alzheimer [12–14]. Accordingly, stool levels in IBDs are routinely measured, thus representing the gold standard to assess inflammation status in patients and providing important hints for clinical exams to make in the follow up (e.g., colonoscopy) [15].

To our knowledge, few studies on circulating CLP have been published in the context of SARS-CoV-2 infection and most of them report serum analysis [16–18]. However, data show that CLP yields on ethylene-diamine-tetra-acetic acid (EDTA) plasma are lower than serum, due to EDTA inhibitory effect on CLP release from neutrophils in vitro [19]. Furthermore, as the release of CLP is strongly accelerated during blood clotting, EDTA plasma is preferable to obtain levels closer to baseline [20].

Currently, inflammatory status assessment relies mainly on few classical tests like C reactive protein (CRP) and erythrocyte sedimentation rate (ESR). In this scenario, an inflammatory markers algorithm which grants information about expected detrimental effects and which retains prognostic value in most of diseases is desirable. CLP feasibility as a standard test in laboratory repertoire is supported by evidence of sustained employment in gastrointestinal inflammation and consistent reports of plasma dosage in autoimmune diseases such as rheumatoid arthritis [10]. Therefore, CLP can be ascribed as a candidate biomarker in a set of laboratory analysis for the inflammation status evaluation, thus also providing insights about improving risk stratification strategies in the plethora of inflammation-driven diseases.

Under these circumstances, SARS-CoV-2 pandemic raised the need to have a battery of preliminary serological tests that can be prognostic for positivity to nasopharyngeal swab regardless the severity of symptoms. Indeed, in situations where is not possible to perform swab tests, the score provided by reliable analytes can improve management of patients and may indicate subsequent diagnostic investigation. In this retrospective observational study, we detected circulating calprotectin (cCLP) levels in patients with confirmed positive nasopharyngeal swab RT-PCR compared to patients with confirmed negative nasopharyngeal swab RT-PCR, as well as healthy controls, to evaluate the clinical outcome predictivity, thus suggesting CLP positive correlation with COVID-19 onset.

## Materials and methods

### Patients and specimens

Samples were recovered, in accordance with local ethical approvals (R.S.44.20), from “Tor Vergata” University Covid-Hospital of Rome as follows:

Serum samples

- 69 negative SARS-CoV-2 RT-PCR subjects (mean age 41.7 years ± 11.1 years; 36 males and 33 females) collected from Tor Vergata Hospital physicians and healthcare workers screened for internal surveillance (Negative RT-PCR controls)
- 71 hospitalized patients with positive SARS-CoV-2 RT-PCR (mean age 67.3 years ± 16.6 years; 33 males and 38 females), collected on days 1–41 from first access to Emergency Department (Positive RT-PCR patients)

Plasma samples

- 71 healthy individuals screened for routine serology analysis (mean age 57.6 years ± 14.9 years; 32 males and 39 females) (Negative controls)
- 59 hospitalized patients with negative SARS-CoV-2 RT-PCR (mean age 61.3 years ± 18.7 years; 29 males and 30 females), collected on the same day of first access to Emergency Department (Negative RT-PCR patients)
- 65 hospitalized patients with positive SARS-CoV-2 RT-PCR (mean age 66.7 years ± 12.8 years; 34 males and 31 females), collected on the same day of first access to Emergency Department (Positive RT-PCR patients)

### Real-time polymerase chain reaction (RT-PCR)

Nasopharyngeal swabs were tested for SARS-CoV-2 infection with Seegene AllplexTM2019-nCoV Assay (Seegene, Seoul, South Korea), according to the manufacturer protocols. Automated RNA extraction and PCR setup were carried out using Seegene NIMBUS, a liquid handling workstation. RT-PCR was run on a CFX96TMDx platform (Bio-Rad Laboratories, Inc., CA, USA) and subsequently interpreted by Seegene’s Viewer Software. The Seegene AllplexTM2019-nCoV Assay identifies the virus by multiplex real-time PCR targeting three viral genes (E, RdRP and N), thus complying with international validated testing protocols.

### Calprotectin chemiluminescence immunoassay (CLIA)

The “QUANTA Flash Calprotectin” kit (INOVA Diagnostics Inc., San Diego, CA, USA) is a chemiluminescent immunoassay for the quantitative calprotectin determination in human samples, performed on the fully automated BIO-FLASH Instrument (INOVA Diagnostics Inc., San Diego, CA, USA), at 37 °C. The principle of the test is a “sandwich immunoassay” where calprotectin-specific capture antibodies are coated on paramagnetic beads. An aliquot of patient sample, antibody-coupled beads and assay buffer are combined into a cuvette. The beads are then magnetized, washed several times until isoluminol-conjugated anti-calprotectin antibodies are added, developing a luminescent reaction when “trigger” reagents are mixed to the cuvette. The light produced from this reaction is measured as Relative Light Units (RLUs) by the BIO-FLASH optical system. RLU values are proportional to the amount of bound isoluminol conjugate, which in turn is proportional to the amount of calprotectin captured on the surface of the beads. By running three calibrators, an instrument specific working curve is created, which is used by the software to calculate ng/ml values from the RLU obtained for each sample.

### Inflammation markers

High-sensitivity C reactive protein (hsCRP) was determined in serum by a latex immunoassay for quantitative immunoturbidimetric detection with a commercial kit performed on the Architect c16000 analyzer (“CRP Vario” assay, Abbott Laboratories, Chicago, USA). The hsCRP levels were reported in mg/L (reference range 0–5 mg/L).

Fibrinogen quantitative determinations in human citrated plasma samples were performed on the ACL Top 550 CTS Coagulation analyzer (IL-Instrumentation Laboratory, Bedford, USA), using the Clauss method based on the fibrinogen ability to form fibrin clot after being exposed to a high concentration of purified thrombin and by comparing sample thrombin clotting time against a plasma standard (reference range 238–498 mg/dL).

Erythrocyte sedimentation rate (ESR) measurements were performed by capillary photometric‐kinetic technology on the fully automated Test 1 SDL analyzer (Alifax S.p.A., Padova, Italy). Whole blood samples are delivered into a capillary tube where they are accelerated via a “stopped‐flow” circuit, which causes sedimentation of erythrocytes. Results are transformed to Westergren values (reference range: males 2–25 mm/h, females 2–30 mm/h).

IL-6 levels (reference range: 0–50 pg/mL) were measured by an automated chemiluminescent assay on the Immulite 2000 instrument (Siemens, Milan, Italy). TNF-α levels (reference range: 0–12.4 pg/mL) were detected by an automated enzyme-linked immunosorbent assay (ELISA) performed on the Immunomat analyzer (Institut Virion/Serion, Wurzburg, Germany).

White blood cell (WBC) count was determined by an automated hematological analyzer (Dasit-Sysmex, Milan, Italy). We also determined the neutrophil-to-lymphocyte ratio (NLR) as a marker of systemic inflammation.

### Statistical analysis

Results were calculated by Mann–Whitney test. Statistical significance was defined as *p* < 0.05. Specificity and sensitivity were calculated by Receiver Operating Characteristic Curves (ROC Curve). All data were analyzed using GraphPad Prism Software 8.0.1 (San Diego, California, USA). The investigators were blinded to the group allocation during the experiment.

## Results

**Fig. 1:**
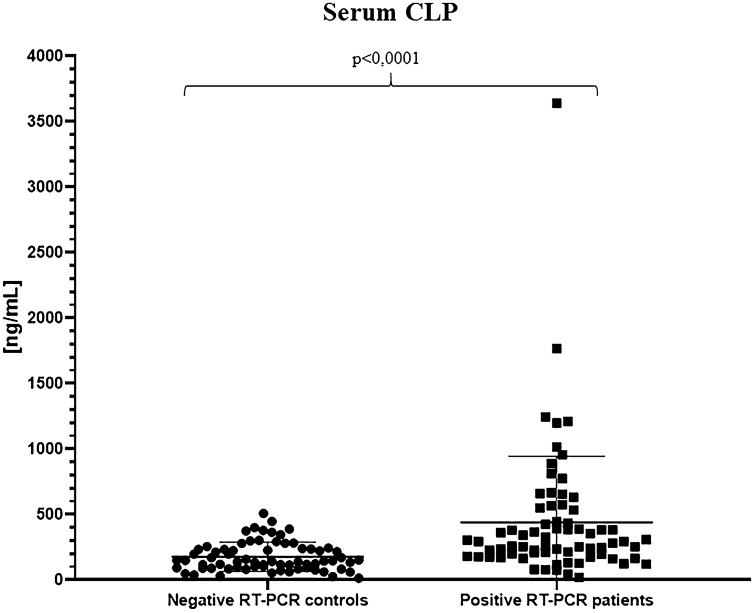
Serum calprotectin concentrations in the negative SARS-CoV-2 RT-PCR control group and in the positive SARS-CoV-2 RT-PCR hospitalized COVID-19 patient group

To evaluate cCLP as a reliable marker of inflammation in the context of SARS-CoV-2 infection, we initially compared hospitalized COVID-19 patients to healthcare workers screened negative to nasopharyngeal swab for SARS-CoV-2, by analyzing cCLP levels in serum. As expected, our cohort shows a statistically significant difference between the two groups, thus confirming the increase of calprotectin median concentration in serum of patients with COVID-19 (292.7 ng/ml; range 19.13–3642 ng/ml) vs negative RT-PCR control group (143.9 ng/ml; range 13.91–508.4 ng/ml) (Table 1; Fig. 1).

**Table 1:** Median and mean circulating calprotectin concentration. Serum calprotectin was detected on negative RT-PCR controls and positive RT-PCR patients; plasma calprotectin was detected on healthy controls, negative RT-PCR patients and positive RT-PCR patients

	Patients’ cohort	Mean [CLP]	Std. deviation	Median [CLP] (range)
Serum	Negative RT-PCR controls (*N* = 69)	176.9 ng/mL	± 110.7 ng/mL	143.9 ng/mL (13.91–508.4)
	Positive RT-PCR patients (*N* = 71)	439.9 ng/mL	± 504.0 ng/mL	292.7 ng/mL (19.13–3642)
Plasma	Negative controls (*N* = 71)	45.36 ng/mL	± 31.13 ng/mL	32.87 ng/mL (14.00–152.20)
	Negative RT-PCR patients (*N* = 59)	177.2 ng/mL	± 201.5 ng/mL	104.8 ng/mL (32.17–1022)
	Positive RT-PCR patients (*N* = 65)	352.3 ng/mL	± 371.6 ng/mL	222.7 ng/mL (48.35–1621)

Later on, considering that cCLP stability is established by evaluation on plasma, we took advantage of samples collected from patients’ first access to Emergency Department resulted positive or negative to nasopharyngeal swab RT-PCR for SARS-CoV-2, exploiting as control group healthcare workers and outpatient users from Tor Vergata Hospital with negative inflammation markers (i.e., CRP < 0.1 mg/L, ESR < 2 mm/h).

**Fig. 2:**
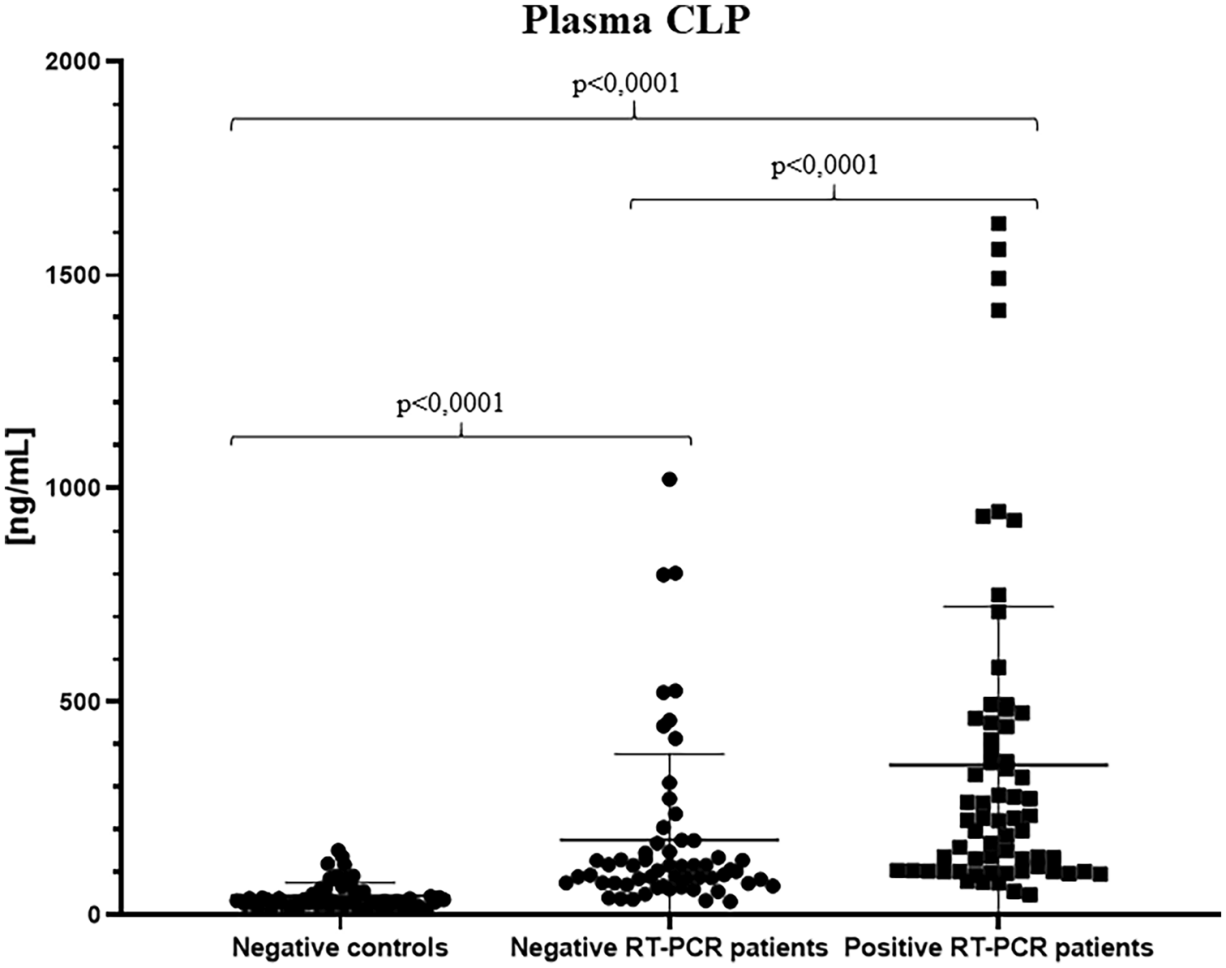
Plasma calprotectin concentrations in the healthy negative control group, in the negative SARS-CoV-2 RT-PCR patient group and in the positive SARS-CoV-2 RT-PCR patient group

Statistical analysis on calprotectin values provided interesting significant differences among the groups: overall median results confirmed cCLP increase in plasma of patients who tested positive for nasopharyngeal swab (222.7 ng/ml; range 48.35–1621 ng/ml), respect to patients with negative swab (104.8 ng/ml; range 32.17–1022 ng/ml) and to the control group (32.87 ng/ml; range 14.00–152.20 ng/ml) (Table 1; Fig. 2).

**Fig. 3:**
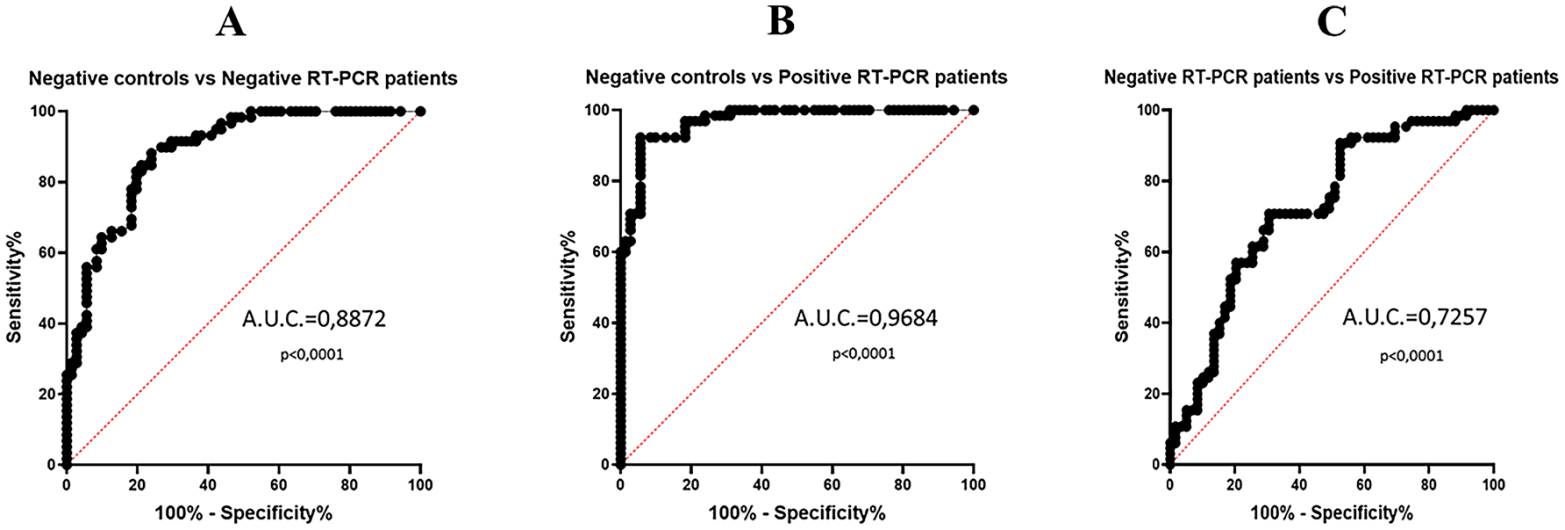
Plasma calprotectin ROC curves. Panel A shows the comparison between healthy negative control group and negative SARS-CoV-2 RT-PCR patient group (AUC = 0.8872, *p* < 0.0001); panel B shows the comparison between healthy negative control group and positive SARS-CoV-2 RT-PCR patient group (AUC = 0.9684, *p* < 0.0001); panel C shows the comparison between negative SARS-CoV-2 RT-PCR patient group and positive SARS-CoV-2 RT-PCR patient group (AUC = 0.7257, *p* < 0.0001)

Based on this excellent evidence, we calculated specificity and sensitivity using receiver-operating characteristic curves (ROC curves). Comparing controls and patients with negative RT-PCR, an area under curve (AUC) value of 0.8872 was achieved, with a sensitivity of 83.05% and a specificity of 80.28% at a cutoff value of 76.45 ng/ml. Intriguingly, matching controls with the positive RT-PCR group, we obtained an AUC value of 0.9684, with a sensitivity of 92.31% and specificity of 94.37% at a cutoff value of 93.04 ng/ml. Finally, the ROC curve resulting from negative and positive RT-PCR group comparison shows an AUC value of 0.7257 with a specificity of 70.77% and a sensitivity of 69.49% at a cutoff value of 131.3 ng/ml, thus suggesting that cCLP can be considered as an inflammation marker able to discriminate between negative and positive patients to SARS-CoV-2 infection, with the best fit cutoff that emerged from our data analysis (Table 2; Fig. 3).

**Table 2:** Plasma calprotectin sensitivity, specificity, cutoff and AUC values obtained in the different group comparisons (healthy controls vs negative RT-PCR patients; healthy controls vs positive RT-PCR patients; negative RT-PCR patients vs positive RT-PCR patients)

	Negative controls vs negative RT-PCR patients	Negative controls vs positive RT-PCR patients	Negative RT-PCR patients vs positive RT-PCR patients
Sensitivity	83.05%	92.31%	70.77%
Specificity	80.28%	94.37%	69.49%
Cutoff	> 76.45 ng/mL	> 93.04 ng/mL	> 131.3 ng/mL
Area under ROC curve (AUC); 95% confidence interval	0.8872; 0.8328–0.9416	0.9684; 0.9443–0.9924	0.7257; 0.6358–0.8156

Since cCLP has been suggested as a reliable biomarker of inflammation in several diseases, we first tested the correlation with two parameters frequently increased in most of COVID-19 longitudinal studies, like CRP and fibrinogen (FBG) by means of Pearson’s test. Accordingly, cCLP correlates positively with CRP and FBG (*r *= 0.69: *r* = 0.7, respectively) in negative swab patients; the correlation is maintained in positive swab patients even though to a lesser extent (*r* = 0.366; *r* = 0.378).

We have also retrospectively analyzed IL-6 and TNF-α levels, known to be involved in the “cytokine storm”. Based on the tests requested by the clinicians at the same time as the assessment of calprotectin levels, we found results only in 35 out of 65 positive RT-PCR patients and no data on negative RT-PCR patients: IL-6 levels were over threshold in 19 patients (median 57.2 pg/mL; range 2.42–5000 pg/mL); TNF-α levels were over threshold in 13 patients (median 7.55 pg/mL; range 1.78–142 pg/mL) and only 10 out of 35 patients were positive for both IL-6 and TNF-α.

Moreover, since calprotectin represents 60% of neutrophils’ granules content, we could reasonably suggest that the calprotectin analyzed in plasma was of neutrophil derivation. To this end, we retrospectively analyzed the neutrophil and lymphocyte counts. As expected, our cohort showed a median neutrophil count significant increase in positive RT-PCR patients (8.35 × 10^3^/µL; range 2.3–43.69 × 10^3^/µL) compared to negative RT-PCR patients (5.69 × 10^3^/µL; range 0.66–24.2); *p* value = 0.0123. Conversely, median lymphocyte count showed higher values in negative RT-PCR patients (1.16 × 10^3^/µL; range 0.37–9.49 × 10^3^/µL) than positive RT-PCR patients (0.91 × 10^3^/µL; range 0.06–2.96 × 10^3^/µL); *p* value *p* = 0.0224. In addition, our data showed an increased neutrophil-to-lymphocyte ratio in positive RT-PCR patients compared to negative RT-PCR patients (9.17 vs 4.9, respectively; *p* value = 0.0002), corroborating other studies in which NLR is considered a systemic inflammatory biomarker as well as a prognostic factor in SARS-CoV-2 infection [21, 22].

## Discussion

In the last 20 years, considerable evidence has developed that supports the use of fecal calprotectin as a marker of intestinal inflammation. However, several studies have reported that circulating calprotectin proves to be an excellent marker capable of providing relevant information about subclinical inflammation in patients suffering from diseases such as rheumatoid arthritis and cystic fibrosis [10, 14].

SARS-CoV-2 pandemic in 2020 has put a strain on health systems in several countries with profound repercussions on global economy and societies. In this scenario, an urgent need arises for fast, reliable tests and markers that can support and guide diagnostic responses, giving first important clinical information in SARS-CoV-2-positive patients.

In this observational study, we sought to investigate the efficacy of circulating calprotectin as a marker of inflammation in the context of SARS-CoV-2 infection, exploiting blood samples from patients taken in care at Tor Vergata Hospital COVID Center of Rome. Serum analysis of COVID-19 hospitalized patients showed that circulating calprotectin median levels are significantly higher than control population (143.9 ng/ml vs 292.7 ng/ml, respectively; *p* value < 0.0001), confirming that calprotectin could be routinely used to well discriminate between healthy and pathological patients.

Subsequent studies were focused on circulating calprotectin in plasma from patients who accessed to Emergency Department of Tor Vergata Hospital for suspected SARS-CoV-2 infection. Notably, plasma employment for calprotectin dosage is recommended as the presence of EDTA prevents massive calprotectin release by granulocytes, thus ensuring lower concentrations but closer to real values of circulating protein. As expected, plasma calprotectin has higher median levels in patients with suspected SARS-CoV-2 infection but negative to RT-PCR, when compared to the control group (104.8 ng/ml vs 32.87 ng/ml, respectively; *p* value < 0.0001). Noteworthy, these differences are significantly increased in case of confirmed RT-PCR positivity, showing overall higher median calprotectin values (222.7 ng/ml; *p* value < 0.0001). Calprotectin could be therefore a marker capable to distinguish quite properly between COVID-19-positive and -negative patients. Furthermore, ROC curves results explored the discrimination ability of circulating plasma calprotectin. The comparison between positive RT-PCR patients and negative control group displays that circulating calprotectin is a good inflammatory marker as it affords high percentages of sensitivity and specificity (92.31%, 94.37%; respectively) with an AUC = 0.9684, considering a best fit cutoff value > 93.04 ng/ml. However, the application of this cCLP concentration does not allow to differentiate between patients with confirmed SARS-CoV-2 infection from patients with negative swab tests. Therefore, taking in comparison only samples from patients with suspicion of infection grouped by certified RT-PCR positivity/negativity, we obtained a best fit cutoff value > 131.3 ng/ml, which allows to better distinguish patients infected by SARS-CoV-2. Accordingly, the lowering in sensitivity and specificity (70.77%, 69.49%; respectively) is due to the comparison performed on a patient control group with somewhat ascertained inflammatory status that does not reflect the general population.

Since calprotectin is mainly released from neutrophils’ granules content and neutrophils are the first responders to many infections, we also analyzed the neutrophil and lymphocyte counts according to evidence of their recruitment in the immune response triggered by SARS-CoV-2 [23–25]. Our data showed a higher median neutrophil count in positive RT-PCR patients compared to negative RT-PCR patients, in contrast with a lower median lymphocyte count in positive RT-PCR patients compared to negative RT-PCR patients.

In addition, we found an increased NLR in positive RT-PCR patients compared to negative RT-PCR patients, sustaining the recent literature proposing increased neutrophil counts and lymphocytopenia as typical clinical features in COVID-19 patients and confirming the same trend for the circulating calprotectin levels [26].

Furthermore, it is well known that Toll-Like Receptor (TLR) can be stimulated by calprotectin leading to release of pro-inflammatory cytokines and the so-called “cytokine storm” can develop in severe cases of COVID-19, which exacerbates the damage caused by the infection [5, 27]. In this line, we found IL-6 and TNF-α levels over threshold in 19 and 13 positive RT-PCR patients, respectively. Since, peripheric blood calprotectin is mainly neutrophil-derived and circulating calprotectin can stimulate TLR, leading to expression and release of pro-inflammatory cytokines, it could presumably be considered as an early and more informative marker in the inflammation context compared to the “cytokine storm” [5, 28].

Finally, correlation analysis was positive for C reactive protein and fibrinogen, thus demonstrating that circulating calprotectin acts as an excellent marker in the inflammatory setting.

Moreover, several studies propose circulating calprotectin in the prognosis of different inflammation-elicited diseases, as well as assessing subclinical inflammation in patients [29]; therefore, cCLP should be definitely considered in a panel of markers aimed at investigating the inflammation state.

Summing up, this study is strengthened by a relatively large sample size compared to other research about circulating calprotectin detection in the context of SARS-CoV-2 infection and is the first to our knowledge that explores calprotectin performance using plasma, as well as serum samples, from infected patients.

These all, add up to other remarkable insights proposing circulating calprotectin as a new, quantitative and convenient marker in the assessment of inflammatory diseases.

Besides, since CLP assessment is currently mostly applied to inflammatory bowel diseases, it could be relevant to investigate the relationship between fecal and circulating CLP in COVID-19 patients with gastrointestinal manifestations for a further important evidence of its diagnostic value as a promising marker in COVID-19.

Unfortunately, our work has some limitations. The population of the study has been chosen on the basis of available samples collected on the same day of first access to Emergency Department. As a consequence, we had no information regarding the presence of concomitant diseases. Therefore, in light of the aim of the study, RT-PCR positivity/negativity outcome was the only inclusion/exclusion criterion considered.

Furthermore, according to hospital data access policy, we cannot provide any further information on medical records and on the clinical status of the patients, except for laboratory medicine department results.

In conclusion, our data suggest that circulating calprotectin along with being of interest as a generic inflammatory marker may provide important indications in the context of SARS-CoV-2 infection.

Of course, additional studies are required to confirm circulating plasma calprotectin assay as a support for the diagnosis of SARS-CoV-2 infection by larger samples size, taking advantage of longitudinal and retrospective studies.
